# Health Literacy in Pregnant Women and Its Associations with Personal, Socioeconomic, and Health-Related Factors in Primary Care

**DOI:** 10.3390/nursrep15120436

**Published:** 2025-12-08

**Authors:** Evaristo Iván Vicente-Díaz, Myriam Alvariñas-Villaverde

**Affiliations:** 1 Álvaro Cunqueiro University Hospital, 36312 Vigo, Pontevedra, Spain; evaristo.ivan.de.vicente.diaz@sergas.es; 2 Research Group on Education, Physical Activity and Health (GIES10), Galicia Sur Health Research Institute (IIS Galicia Sur), SERGAS-UVIGO, 36312 Vigo, Pontevedra, Spain; 3 Department of Special Didactics, Universidade de Vigo, 36310 Vigo, Pontevedra, Spain

**Keywords:** health literacy, pregnancy, prenatal care, primary health care, maternal health

## Abstract

**Background/Objectives:** Health literacy (HL) plays a fundamental role in maternal and neonatal outcomes by influencing women’s ability to access, understand, and apply health information during pregnancy. However, evidence regarding the determinants of HL among pregnant women remains limited, particularly within the Spanish context. This study aimed to assess HL levels among pregnant women and to examine their association with personal, socioeconomic, and health-related factors. **Methods:** A cross-sectional study was conducted between January 2023 and February 2024 across nine primary care centres within the Vigo Health Area (Spain), including 182 pregnant women receiving prenatal care. HL was measured using the 16-item European Health Literacy Survey Questionnaire (HLS-EU-Q16). Sociodemographic, obstetric, and health-related variables were collected through structured interviews. Descriptive and inferential analyses were performed to explore associations between HL and the selected variables. **Results:** Limited HL was observed in 35.7% of participants. A significant association was found between HL and family income (*p* = 0.037), with limited HL being more frequent among women with a monthly family income below €2000. No associations were identified with other sociodemographic or health-related variables. Thirty-nine per cent of participants visited hospital emergency services on two or more occasions, mostly without admission. The main source of information was healthcare professionals, although Internet use was also relevant. **Conclusions:** The prevalence of limited HL was lower than that reported in other national studies, although inequalities related to family income persisted. These findings highlight the need to incorporate systematic, HL-tailored strategies into prenatal care, based on prior HL assessment, to promote informed decision-making and improve maternal and neonatal outcomes.

## 1. Introduction

Health literacy (HL) is defined as the set of knowledge, motivations, and competencies that enable individuals to access, understand, appraise, and apply health-related information in order to make appropriate decisions in the domains of healthcare, disease prevention, and health promotion [1]. From this perspective, HL represents a fundamental requirement for navigating the increasing demands of contemporary healthcare systems and for functioning within information environments characterised by their complexity, multiplicity of sources, and progressive digitalisation [2].

Insufficient HL levels are consistently associated with higher rates of hospitalisation, poorer adherence to treatment regimens, lower engagement in preventive behaviours, worse overall health outcomes, increased mortality, and greater healthcare costs, in addition to contributing to widening health inequalities [3,4]. Given its impact on health equity and population wellbeing, the World Health Organization has recognised HL as a key determinant of health, incorporating its promotion among the strategic pillars of the 2030 Agenda for Sustainable Development and considering it an essential component for strengthening public health globally [4,5].

During pregnancy, HL becomes particularly critical, as this period is marked by heightened health demands, multiple informational needs, and decision-making processes that directly influence both maternal and neonatal wellbeing. Pregnant women must navigate diverse sources of health information, often heterogeneous in quality, while communicating effectively with healthcare professionals, making HL an indispensable resource for safely navigating the gestational process [6].

Inadequate HL during pregnancy has been associated with lower adherence to preventive behaviours, such as delayed initiation of prenatal care, inadequate use of pregnancy-specific supplements, or a reduced likelihood of maintaining exclusive breastfeeding [7,8,9]. It has also been linked to difficulties in reproductive planning, limited involvement in clinical decision-making, and a higher risk of inappropriate use of healthcare services. These limitations may contribute to the development of obstetric complications, including gestational diabetes, hypertension, or maternal depression, as well as adverse neonatal outcomes such as low birth weight and other perinatal health problems [6,10,11].

HL levels during pregnancy show substantial international variability, influenced by cultural, socioeconomic, and educational factors, as well as by the instruments used to assess HL. Reported prevalences range from relatively low values, around 15% in the United States [12], to much higher proportions exceeding 70% in Latin American countries [13]. Elevated levels have also been described in other settings, such as 67% in Iran [14], and intermediate values in African contexts, including 41% in Nigeria [15]. In Europe, although variability is less pronounced, considerable differences persist, with estimates as low as 6.8% in the Netherlands [16], intermediate values around 35% in countries such as Germany [17], and proportions above 45% in southern regions of the continent, including Portugal [18]. In Spain, available evidence on HL in pregnant women remains limited and derives mainly from studies conducted in specific territorial areas of the country, where high proportions of inadequate or limited HL, often above 40%, have been reported [19,20]. However, more recent multicentre research has identified more moderate figures, close to 30% [9], suggesting the existence of variability even within the same healthcare system. This scenario underscores the importance of analysing HL in specific contexts, taking into account the sociodemographic, cultural, and healthcare characteristics of each population.

In this context, the present study aimed to assess HL levels among pregnant women receiving care in the Vigo Health Area and to explore their association with personal, socioeconomic, and health-related factors. Generating data from specific healthcare settings is essential for understanding territorial variability in HL during pregnancy and for supporting the design of strategies aimed at enhancing the comprehension of health information within prenatal care.

## 2. Materials and Methods

### 2.1. Study Design

A cross-sectional observational design was used, appropriate for describing health literacy levels and examining their association with different factors, following the approach employed in several previous studies in pregnant populations [7,21]. A consecutive non-probabilistic sampling strategy was applied, a method widely used in primary care research due to its feasibility and its capacity to include all eligible women during the study period [8,22]. Although this sampling approach may limit the generalisability of the findings beyond the healthcare context examined, it represents a common methodological choice in health literacy research.

### 2.2. Setting

The study was conducted in the Vigo Health Area, one of the seven public healthcare areas in Galicia (north-west Spain), which provides services to approximately 199,500 women of reproductive age. Specialised maternal healthcare is centralised at Álvaro Cunqueiro Hospital, which registers around 3000 births annually. To ensure territorial representativeness, eight geographically distributed primary healthcare centres were selected. Fieldwork was carried out between January 2023 and February 2024.

### 2.3. Participants

Inclusion criteria: adult pregnant women over 30 weeks of gestation, whose delivery was planned at the reference maternity unit of the health area, and who provided written informed consent to participate.

Exclusion criteria: refusal to participate, insufficient oral or written comprehension of Spanish or Galician to ensure proper participation, or incorrectly completed questionnaires.

### 2.4. Data Collection

Participants were recruited during group-based prenatal education sessions held at the selected centres. To ensure consistency in questionnaire administration, collaborating healthcare professionals received specific training on the application procedure, inclusion and exclusion criteria, and the management of frequently asked questions. When the questionnaire was not self-administered directly by the researchers, a standardised protocol was followed to guarantee uniformity in data collection.

Participation was voluntary, following the provision of an information sheet and the signing of informed consent. Questionnaires were completed anonymously in a quiet environment, without the presence of companions, and were returned in sealed envelopes. To safeguard confidentiality, all questionnaires were coded using a non-traceable identification system with no link to personal data. Only the research team had access to the dataset, which was stored and managed in accordance with current data protection legislation.

### 2.5. Variables

Health literacy was assessed using the 16-item version of the European Health Literacy Survey Questionnaire (HLS-EU-Q16) [23], a shortened form of the original instrument developed within the European Health Literacy Survey project [1] and validated in Spain, where it demonstrated a Cronbach’s alpha of 0.982 [24]. The instrument comprises 16 items that measure individuals’ perceived ability to access, understand, appraise, and apply health-related information across three key domains: healthcare, disease prevention, and health promotion. The HLS-EU-Q16 has also been previously used in studies involving pregnant and perinatal populations, supporting its suitability for assessing health literacy in this context [25,26].

Each item is rated on a four-point Likert scale (from very easy to very difficult). The total score categorises respondents into different HL levels [25]: inadequate (0–8), problematic (9–12), and sufficient (13–16). Following previous research [24], the present study applied a dichotomous classification: inadequate HL (0–12) and adequate HL (13–16).

Additional data were collected on personal variables (age, marital status, type of cohabitation, educational level), socioeconomic variables (area of residence, current occupation, personal monthly income, and family monthly income), and health-related variables (hospital admissions, emergency department visits, and main source of health information).

### 2.6. Sample Size

The sample size was estimated using the formula for infinite populations, assuming an expected prevalence of 13% inadequate HL based on previous data [27], a 95% confidence level, and a 5% margin of error. The required sample size was calculated as 174 participants. To account for potential losses, the sample was increased by 5%, resulting in 183 recruited participants. In total, 182 women completed the questionnaire correctly and were included in the analysis.

### 2.7. Statistical Analysis

A descriptive analysis of categorical variables was performed using frequency and percentage distributions. The chi-square test and Fisher’s exact test were used to compare categorical variables. A 95% confidence level was assumed for all analyses. Statistical analyses were conducted using IBM SPSS^®^ Statistics for Windows, version 24.0. The level of statistical significance was set at *p* < 0.05.

## 3. Results

A total of 182 pregnant women participated in the study. The main sociodemographic characteristics are presented in Table 1. The mean age was 34.52 years (SD = 4.44). Most participants were married or living with a partner (91.8%) and nearly half resided in urban areas (48.9%). Higher education was the most frequent level attained (48.4%), and the majority were in paid employment (68.1%). More than two-thirds lived with their partner (68.7%). Personal monthly income most commonly ranged between €1001 and €1500 (51.1%), whereas family income was predominantly between €2001 and €3000 (63.7%).

Regarding healthcare use during pregnancy, only a small proportion required hospital admission (10.4%), yet a considerable number attended emergency services two or more times (39%). Healthcare professionals were the main source of information during pregnancy (64.8%), followed by the Internet (26.9%).

Overall, 64.3% of women presented sufficient health literacy, while 35.7% had limited levels. Within this latter group, 2.2% showed inadequate HL and 33.5% problematic HL.

A statistically significant association was observed between monthly family income and HL level (χ^2^ = 6.597; df = 2; *p* = 0.037) (Table 2). Among women with a monthly family income below €2000, 54.3% presented limited HL, compared with 29.2% and 29.0% in the two higher income categories. Conversely, adequate HL predominated among women with incomes above €2000. A significant linear trend (*p* = 0.028) further indicated that higher household income was associated with higher HL levels.

For the remaining personal, socioeconomic and health-related variables, no statistically significant associations were identified. However, some descriptive variations were observed. For instance, although women with basic education showed the highest proportion of limited HL (50.0%), this pattern was not consistent across groups. Similarly, limited HL appeared slightly more frequent among women living with a partner compared with those living in extended or other family arrangements, and women relying mainly on the Internet tended to show a higher proportion of limited HL than those who consulted healthcare professionals. These variations did not reach statistical significance and should therefore be interpreted with caution.

## 4. Discussion

The proportion of pregnant women with limited health literacy found in our study was lower than that reported in most national [19,20,27] and international [16,17] investigations. Such variations, widely documented in the literature, may be explained by differences in assessment instruments, classification criteria, or the sociodemographic characteristics of the populations studied, which complicate direct comparison across contexts. Nevertheless, although the proportion observed in our sample was slightly higher, our findings align with the recent data reported Vila-Candel et al. [9] who identified 30.5% of limited health literacy using the same instrument. This proximity situates our results within the range currently described in the Spanish context.

Regarding the determinants analysed, family income was the only factor significantly associated with health literacy. Women from households with fewer economic resources were more likely to present limited health literacy. This pattern is consistent with studies indicating that material conditions and support structures within the household decisively influence the ability to access, understand, and use health information [1,28,29,30]. In contrast, individual income showed no association in our study, a finding that mirrors previous research [13,22,25]. Family income appears to better capture the collective, educational, and structural dynamics that shape decision-making during pregnancy. Taken together, these findings suggest that socioeconomic inequalities operate as structural determinants influencing health equity beyond individual attributes.

By contrast, age, educational level, cohabitation, and employment status showed no significant association with health literacy. Although the literature reveals heterogeneous findings, with some studies identifying higher levels among women aged 25–35 years [19,26,31] and others reporting higher literacy among younger women with greater digital competence [32,33], the broader evidence suggests that age functions more as a moderating factor linked to reproductive experience and digital skills than as a direct determinant. A similar pattern is observed for education. Although numerous studies describe it as a key determinant [34,35,36], other authors highlight that higher academic attainment does not necessarily guarantee adequate health literacy, as functional skills such as reading comprehension, critical reasoning, or digital proficiency may vary considerably among individuals with comparable educational levels [37,38,39]. With regard to cohabitation, our findings align with studies reporting no direct association [9], despite evidence elsewhere suggesting potential protective effects related to emotional or communicative support within the household [22,40,41]. Overall, these results point to the influence of multicausal dynamics that extend beyond traditional sociodemographic variables.

No significant associations were detected between health literacy and healthcare service use. Although previous literature has reported more frequent emergency visits and longer hospital stays among individuals with limited health literacy [37,42,43], our findings did not reproduce this pattern. This may reflect the universal and accessible nature of the Spanish healthcare system, together with structured prenatal follow-up programmes that tend to homogenise service utilisation irrespective of health literacy levels. Nonetheless, the high proportion of women who attended emergency services two or more times (39%) suggests the need to examine non-clinical factors such as insecurity, persistent doubts, or difficulties recognising warning signs, which may influence help-seeking behaviour [44]. Furthermore, although no significant differences were found regarding the main source of information, the increasing use of the Internet observed in our sample aligns with previous studies [8] and underscores the importance of strengthening critical appraisal skills in digital environments.

These findings carry relevant implications for clinical practice. First, they highlight the importance of assessing health literacy individually during prenatal follow-up, avoiding assumptions based solely on age, education, or employment status. Second, the association with family income suggests that primary care teams should identify women experiencing socioeconomic vulnerability at an early stage and offer tailored support measures such as accessible educational materials, reinforcement sessions, or community-based interventions. In addition, the growing use of the Internet as a source of information reinforces the need for midwives and obstetricians to guide pregnant women towards reliable and culturally appropriate resources, promoting critical skills to navigate information overload. Finally, the high frequency of emergency department visits indicates the value of strengthening antenatal education on warning signs and doubt resolution, thereby supporting pregnant women’s autonomy and safety.

## 5. Limitations

This study has several limitations that should be considered when interpreting the findings. First, the cross-sectional design does not allow causal relationships to be established between health literacy levels and the variables analysed. Second, although consecutive sampling is commonly used in health literacy research and is appropriate in clinical settings, it limits the generalisability of the results because the sample was drawn from a single health area. Additionally, although the HLS-EU-Q16 enabled a standardised assessment, data were collected through a self-administered questionnaire, which may introduce social desirability or comprehension biases. The study did not incorporate certain potential determinants of health literacy, which should be addressed in future research. Finally, because only one variable showed a significant association in the bivariate analysis, it was not possible to conduct a multivariate analysis to further explore the potential influence of confounding factors.

Despite these limitations, the study provides valuable evidence regarding health literacy during pregnancy in a territorially underrepresented context and offers useful insights for guiding tailored interventions and future research.

## 6. Conclusions

The findings of this study indicate that although pregnant women in the Vigo Health Area generally display adequate levels of HL, inequalities related to family income persist. This result reinforces the influence of social and economic determinants in the perinatal context and highlights the need to address them comprehensively to ensure equitable, woman-centred care.

Moreover, the importance of consolidating reliable, accessible, and HL-adapted information channels, as well as enhancing healthcare professionals’ communication skills, is underscored. The systematic incorporation of strategies based on prior HL assessment into prenatal care could promote informed decision-making, adherence to recommendations, and improved maternal and neonatal outcomes.

Finally, further multicentre and longitudinal research is warranted to explore the factors influencing HL during pregnancy and to evaluate the effectiveness of interventions designed to reduce the identified inequalities and optimise the quality of prenatal care.

## Figures and Tables

**Table 1:** Sociodemographic characteristics of the participants (n = 182).

Variable	n	%
Age		
>35 years	82	45.1
≤35 years	100	54.9
Marital status		
Single	14	7.7
Married	167	91.8
Divorced	1	0.5
Living arrangements		
With partner	125	68.7
With partner and children/other relatives	49	26.9
Others	8	4.4
Area of residence		
Rural	42	23.1
Semi-rural	51	28.0
Urban	89	48.9
Educational level		
Basic	26	14.3
Intermediate	68	37.4
Higher	88	48.4
Current occupation		
Salaried employment	124	68.1
Self-employed	28	15.4
Receiving benefits/unemployed/unpaid work	30	16.5
Monthly personal income (€)		
<1000	46	25.3
1001–1500	93	51.1
>1500	43	23.6
Monthly family income (€)		
<2000	35	19.2
2001–3000	116	63.7
>3000	31	17.0
Number of hospital admissions		
None	163	89.6
1–2 admissions	19	10.4
Number of emergency service visits		
None	38	20.9
Once	73	40.1
Twice	50	27.5
Three or more times	21	11.5
Main source of information during pregnancy		
Midwife	98	53.8
Gynaecologist	20	11.0
Internet	49	26.9
Others	15	8.2

Note: Data are presented as n (%).

**Table 2:** Health literacy level according to personal, socioeconomic, and health-related parameters (n = 182).

Variable	Limited HL, n (%)	Adequate HL, n (%)	p Value
Age			
>35 years	27 (32.9)	55 (67.1)	0.477
≤35 years	38 (38.0)	62 (62.0)	
Marital status			
Without partner (single/divorced/widowed)	5 (33.3)	10 (66.7)	0.841
Married or with partner	60 (35.9)	107 (64.1)	
Cohabitation			
With partner	20 (40.8)	29 (59.2)	0.421
With partner and children/other relatives	41 (32.8)	84 (67.2)	
Other	4 (50.0)	4 (50.0)	
Residence			
Rural	15 (35.7)	27 (64.3)	0.997
Semi-rural	18 (35.3)	33 (64.7)	
Urban	32 (36.0)	57 (64.0)	
Educational level			
Basic	13 (50.0)	13 (50.0)	0.235
Intermediate	24 (35.3)	44 (64.7)	
Higher	28 (31.8)	60 (68.2)	
Current occupation			
Paid employment	45 (36.3)	79 (63.7)	0.337
Self-employed	7 (25.0)	21 (75.0)	
Unemployed/receiving benefits/unpaid work	13 (43.3)	17 (56.7)	
Monthly income			
<€1000	20 (43.5)	26 (56.5)	0.309
€1001–1500	33 (35.5)	60 (64.5)	
>€1500	12 (27.9)	31 (72.1)	
Monthly family income			
<€2000	19 (54.3)	16 (45.7)	0.037
€2001–3000	37 (31.9)	79 (68.1)	
>€3000	9 (29.0)	22 (71.0)	
Number of hospital admissions			
None	59 (36.2)	104 (63.8)	0.691
1–2 admissions	6 (31.6)	13 (68.4)	
Number of emergency visits			
None	13 (34.2)	25 (65.8)	0.867
Once	28 (38.4)	45 (61.6)	
Twice	18 (36.0)	32 (64.0)	
Three or more times	6 (28.6)	15 (71.4)	
Main source of information during pregnancy			
Midwife	29 (29.6)	69 (70.4)	0.22
Obstetrician-gynaecologist	7 (35.0)	13 (65.0)	
Internet	23 (46.9)	26 (53.1)	
Other	6 (40.0)	9 (60.0)	

Note: Data are presented as n (%). Statistical significance was set at p < 0.05.

## Data Availability

The data presented in this study are available on reasonable request from the corresponding author. The data cannot be publicly shared due to ethical and legal requirements, as the dataset contains sensitive personal health information and confidentiality must be protected.

## Public Involvement Statement

No public involvement in any aspect of this research.

## Guidelines and Standards Statement

This manuscript adheres to the STROBE (Strengthening the Reporting of Observational Studies in Epidemiology) guidelines for observational cross-sectional studies.

## Abbreviations

The following abbreviations are used in this manuscript:

HL	Health literacy
HLS-EU-Q16	European Health Literacy Survey Questionnaire (16-item version)
WHO	World Health Organization
